# Control of Line Complications with KiteLock (CLiCK) in the critical care unit: study protocol for a multi-center, cluster-randomized, double-blinded, crossover trial investigating the effect of a novel locking fluid on central line complications in the critical care population

**DOI:** 10.1186/s13063-022-06671-5

**Published:** 2022-08-30

**Authors:** Marlena Ornowska, Hubert Wong, Yongdong Ouyang, Anish Mitra, Aaron White, Sue Willems, Jessica Wittmann, Steven Reynolds

**Affiliations:** 1 Simon Fraser University, Burnaby, Canada; 2 Center for Health Evaluation and Outcome Sciences, University of British Columbia, Vancouver, Canada; 3 University of British Columbia, Vancouver, Canada; 4 Fraser Health Authority, Surrey, Canada; 5 Vaccine and Infectious Disease Organization, University of Saskatchewan, Saskatoon, Canada

**Keywords:** Central venous access device, Catheter locking, Intensive care unit, Central line-associated bloodstream infection, Catheter colonization, Central line occlusion, Catheter-related venous thrombosis, Alteplase, Biofilm, Clinical trial

## Abstract

**Background:**

Insertion of a central venous access device (CVAD) allows clinicians to easily access the circulation of a patient to administer life-saving interventions. Due to their invasive nature, CVADs are prone to complications such as bacterial biofilm production and colonization, catheter-related bloodstream infection, occlusion, and catheter-related venous thrombosis. A CVAD is among the most common interventions for patients in the intensive care unit (ICU), exposing this vulnerable population to the risk of nosocomial infection and catheter occlusion. The current standard of care involves the use of normal saline as a catheter locking solution for central venous catheters (CVCs) and peripherally inserted central catheter (PICC) lines, and a citrate lock for hemodialysis catheters. Saline offers little prophylactic measures against catheter complications. Four percent of tetrasodium ethylenediaminetetraacetic acid (EDTA) fluid (marketed as KiteLock Sterile Locking Solution™) is non-antibiotic, possesses antimicrobial, anti-biofilm, and anti-coagulant properties, and is approved by Health Canada as a catheter locking solution. As such, it may be a superior CVAD locking solution than the present standard of care lock in the ICU patient population.

**Methods:**

Our team proposes to fill this knowledge gap by performing a multi-center, cluster-randomized, crossover trial evaluating the impact of 4% tetrasodium EDTA on a primary composite outcome of the incidence rate of central line-associated bloodstream infection (CLABSI), catheter occlusion leading to removal, and use of alteplase to resolve catheter occlusion compared to the standard of care. The study will be performed at five critical care units.

**Discussion:**

If successful, the results of this study can serve as evidence for a shift of standard of care practices to include EDTA locking fluid in routine CVAD locking procedures. Completion of this study has the potential to improve CVAD standard of care to become safer for patients, as well as provides an opportunity to decrease strain on healthcare budgets related to treating preventable CVAD complications. Success and subsequent implementation of this intervention in the ICU may also be extrapolated to other patient populations with heavy CVAD use including hemodialysis, oncology, parenteral nutrition, and pediatric patient populations. On a global scale, eradicating biofilm produced by antibiotic-resistant bacteria may serve to lessen the threat of “superbugs” and contribute to international initiatives supporting the termination of antibiotic overuse.

**Trial registration:**

ClinicalTrials.gov NCT04548713, registered on September 9th, 2020.

**Supplementary Information:**

The online version contains supplementary material available at 10.1186/s13063-022-06671-5.

## Administrative information

Note: The numbers in curly brackets in this protocol refer to SPIRIT checklist item numbers. The order of the items has been modified to group similar items (see http://www.equator-network.org/reporting-guidelines/spirit-2013-statement-defining-standard-protocol-items-for-clinical-trials/).

**Table:** 

Title {1}	Control of Line Complications with KiteLock (CLiCK) in the intensive care unit: study protocol for a multi-center, cluster-randomized, double-blinded, crossover trial investigating the effect of a novel locking fluid on central line complications in the critical care population
Trial registration {2a and 2b}.	ClinicalTrials.gov - NCT04548713, registered on September 9th, 2020.
Protocol version {3}	Protocol Version 2.3 02/2022
Funding {4}	SterileCare Inc., Mitacs IT18412, Royal Columbian Hospital Foundation Grant (RCHFG), CANHealth Network
Author details {5a}	Marlena Ornowska- Simon Fraser University Dr. Hubert Wong- University of British Columbia, Center for Health Evaluation and Outcome Sciences Dr. Yongdong Ouyang- University of British Columbia, Center for Health Evaluation and Outcome Sciences Dr. Anish Mitra- University of British Columbia, Fraser Health Authority Dr. Aaron White- University of Saskatchewan, Vaccine and Infectious Disease Organization Sue Willems- Fraser Health Authority Jessica Wittmann- Fraser Health Authority Dr. Steven Reynolds- Simon Fraser University, Fraser Health Authority
Name and contact information for the trial sponsor {5b}	Sponsor: SterileCare Inc. Karen Mueller, CEO 15 Allstate Parkway, Suite 600 Markham, ON, Canada L3R 5B4 Telephone: 1-844-860-5900 e-mail:kmueller@sterilecareinc.com
Role of sponsor {5c}	This is an investigator-initiated study. Decisions about study design, data collection, management, analysis, and interpretation were made independently of the sponsor and all funders. The study sponsors and funders will not contribute to nor have any authority over data collection, management analysis, and interpretation. The study sponsors and funders will not contribute to, nor have any authority over, writing the report and the decision to submit the report for publication.

## Introduction

### Background and rationale {6a}

Insertion of a central venous access device (CVAD) is an essential medical intervention, allowing clinicians to access the circulation of a patient and administer life-saving interventions. Due to their invasive nature, CVADs are prone to complications such as occlusion, and bacterial colonization of the CVAD that may travel to the bloodstream and cause infection, known as central line-associated bloodstream infection (CLABSI). A CVAD is among the most common interventions in the intensive care unit (ICU) [1] exposing this vulnerable population to potential hospital-acquired infections and CVAD occlusion.

A recent systematic review identified pooled estimates for CLABSI at 4.59 per 1000 catheter days based on calculations from global databases and studies in adult ICUs [2]. This is well above the global target of zero [3]. Significant risk factors associated with developing CVAD-related infection include longer duration of CVAD insertion, administration of total parenteral nutrition, presence of multiple CVADs, and use of CVADs that have multiple lumens [4]. Unfortunately, all of these risk factors are common among ICU patients and often cannot be modified due to clinical needs. It is also known that bacteria that colonize the CVAD aggregate into microcolonies known as biofilm, which exhibit different properties than single units of bacteria and result in a much more difficult to treat infection [5].

In addition to posing a significant risk to the patient, hospital-acquired bloodstream infections are associated with an increased median length of stay of 2 days and a $12,321 increase per case costs in Canadian ICUs, when compared to matched-cohort controls [6]. The most recent report by the Canadian Nosocomial Infection Surveillance Program states a combined incidence rate of 159 CVAD-associated bloodstream infections diagnosed annually in the 54 participating hospitals, costing the healthcare system an estimate of $2,000,000 each year [7]. As not all Canadian hospitals contribute to this reporting, and CLABSI diagnosis criteria may vary between hospitals, this is likely an underestimation of the total cost to the Canadian healthcare system.

In addition to bacterial colonization and infection, CVADs are prone to the formation of thrombi (blood clots) that may occlude the CVAD [5, 8, 9]. A CVAD may also become occluded due to an accumulation of precipitate from medication or parenteral nutrition, or a build-up of biofilm [10, 11]. CVAD occlusion is common, affecting approximately between 14 and 36% of all CVADs [12]. In addition to interrupting treatment through the CVAD, a thrombus may dislodge into the bloodstream and cause further adverse events such as a thromboembolism, or spread an attached bacterial biofilm into the bloodstream [5, 8, 9]. Current treatment of catheter occlusions involves the use of costly thrombolytic agents such as alteplase—the cost of which has more than doubled between 2005 and 2014 and is now priced at $63.40 per dose  [13]. Although expensive, Ernst and colleagues determined treatment with alteplase to cost an average of $317 less per patient compared to complete catheter replacement [12]. As such, effective prevention of CVAD occlusion provides an opportunity to significantly decrease the cost and morbidity associated with ICU care.

Appropriate CVAD care is paramount in preventing infection and occlusion. The current standard of care in the intensive care unit (ICU) involves the use of salt water (saline) as a locking solution for central venous catheters (CVCs) and peripherally inserted central catheters (PICC lines). Although adequate in maintaining patency, saline offers no anti-bacterial or anticoagulant properties that may act as prophylactic measures against infection and occlusion. Hemodialysis lines are locked with citrate. Evidence from the literature shows alternative locking solutions with additional protective properties such as the use of antibiotic, heparin, citrate, or ethanol locks are not efficacious and are costly in terms of unnecessary patient risk, the propagation of antibiotic overuse, and excess cost to the healthcare system [14–22].

Successful prevention of catheter complications requires simultaneous inhibition of bacterial colonization, biofilm formation, and thrombus formation. Four percent of tetrasodium ethylenediaminetetraacetic acid (EDTA) is a fluid that possesses non-antibiotic, anti-microbial, anti-biofilm, and anti-thrombotic properties [23, 24]. As such, it may serve to fill this need when investigated as a locking fluid. Four percent of EDTA (known as “KiteLock 4% Sterile Catheter Lock Solution”) is already approved by Health Canada as a catheter lock solution. In the laboratory environment, microbial biofilm isolates from 12 common CVAD colonizing bacterial and fungal species were completely eradicated by 4% EDTA [25]. Clinical investigations have shown 4% EDTA to reduce CVAD complications in pediatric intestinal failure patients and hemodialysis patients [26–28]. The results of these studies demonstrate a decrease in CLABSI rates from 2.7 to 0 cases per 1000 catheter days and from 1.9 to 0.6 per 1000 catheter days after 12 months of use [27, 28]. Four percent of EDTA has not yet been studied in the particularly vulnerable intensive care population. We propose to conduct a multi-center study to assess the efficacy of 4% EDTA in preventing CVAD infection and occlusion when used as a locking fluid. If successful, this intervention may serve to reduce the risk associated with ICU stay, thus contributing to reduced morbidity and mortality. Furthermore, this intervention may serve to reduce the cost associated with CVAD complication treatment and contribute to worldwide efforts to lessen reliance on antibiotics.

## Objectives {7}

### Primary objectives

The primary objective is to compare the incidence rate of CLABSI, catheter occlusions requiring replacement, and alteplase use present in critical care patients with CVADs locked with 4% tetrasodium EDTA versus critical care patients with CVADs locked with the standard of care of saline or citrate.

For the purpose of this study, these outcomes will be evaluated using a composite outcome consisting of the incidence rate of catheter obstruction requiring alteplase, CLABSI (diagnosed in accordance with NHSN definitions [29]), or CVAD replacement due to occlusion. As current surveillance guidelines do not include routine CVAD occlusion reporting, the use of alteplase specifically for the treatment of CVAD occlusion has been identified as a proxy method to track the occlusion rates.

The secondary objectives include the incidence rate of each component of the composite outcome separately, and other outcomes that may be modifiable with change to catheter locking practices, including: the incidence of catheter colonization (as identified by the standard clinical and microbiology operating procedures at each institution enrolled in the study), incidence of catheter-associated venous thrombosis, direct cost related to alteplase use for catheter occlusion, other costs related to treatment and diagnosis of relevant CVAD complications, and classification of microbial species isolated from colonized catheters.

### Trial design {8}

**Fig. 1:**
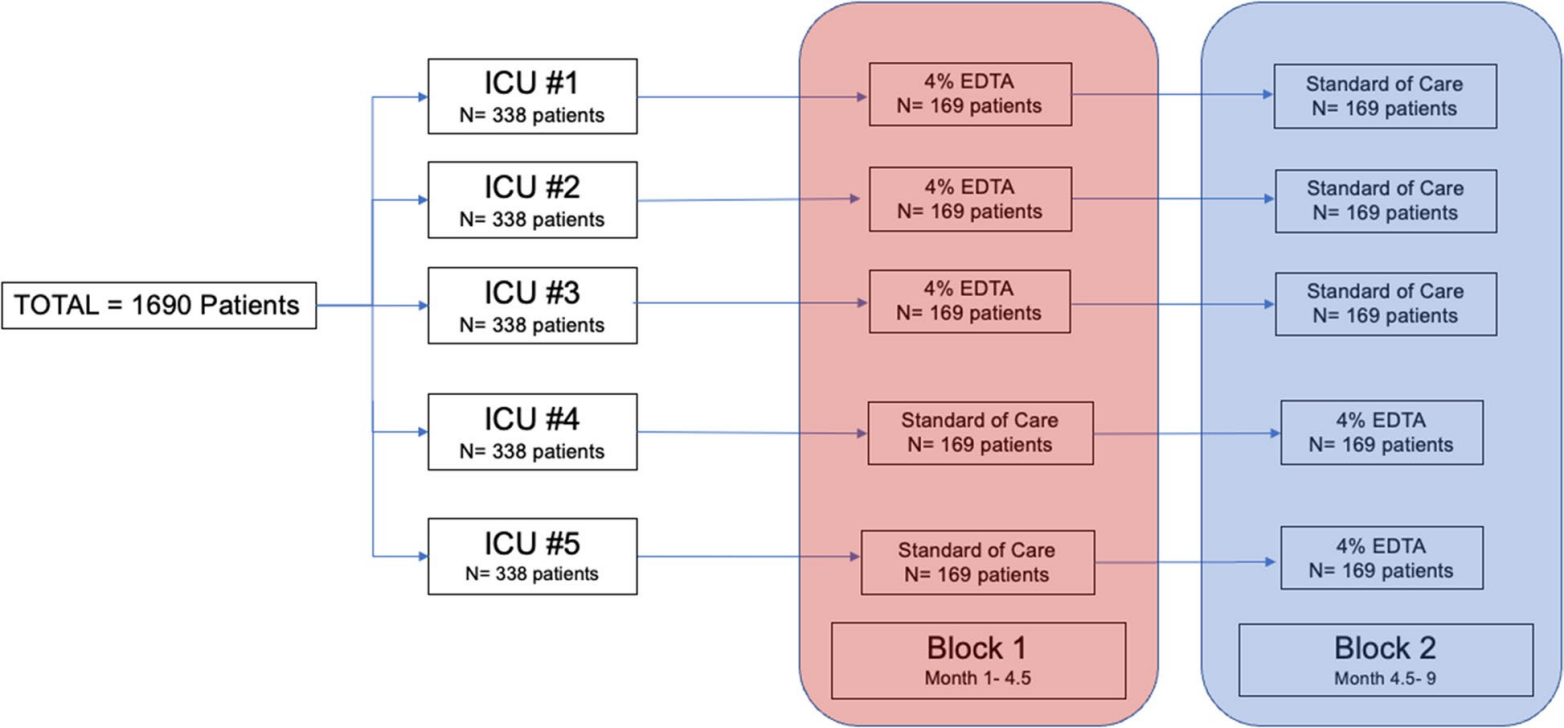
Study protocol

The trial is a multi-center, cluster-randomized, double-blinded, crossover trial. Five ICUs/high acuity units (HAUs) across Canada will participate. The ICU/HAUs will be assigned at random to administer either the 4% EDTA locking solution (intervention) or the standard of care saline or citrate (control) during the first four-and-a-half-month period. ICU/HAUs will then cross over and administer the other locking solution during the second 4.5-month period. Patient recruitment will cease at the end of three-and-a-half months during each period to ensure all patients can be followed for 28 days. The study protocol will commence at each site via a staggered start (see Fig. 1).

## Methods: participants, interventions, and outcomes

### Study setting {9}

This study will take place in five ICU/HAUs in hospitals located across Canada. Participating sites include Royal Columbian Hospital, Surrey Memorial Hospital, Royal Jubilee Hospital, Nanaimo Regional General Hospital, and St. Boniface Hospital.

### Eligibility criteria {10}

The following are the inclusion criteria:

1. ICU/HAU admission.
2. Presence of a central venous catheter requiring locking. This includes triple lumen central venous catheters (both tunneled and non-tunneled), dialysis lines, implanted vascular access devices (IVADs), and peripherally inserted central lines (PICCs).
3. 18 years of age.

The following are the exclusion criteria:

1. Known sensitivity to EDTA.
2. Confirmed or suspected pregnancy, as 4% tetrasodium EDTA has not yet been investigated for use in the pregnant population.
3. Patients who decline receiving blood products (due to the smaller blood draws and special blood conserving procedures).
4. Physician, patient, or temporary substitute decision-maker (TSDM) declines.
5. Currently enrolled in any other research study that may confound primary outcome measures. Co-enrollment in multiple studies will be considered on an individual basis.
6. Patients who were previously enrolled in the study. Patients who were enrolled in the first period are not eligible for (re-)enrollment in the second period, and patients who are enrolled in the study and transferred to another participating hospital are not eligible for (re-)enrollment at the receiving hospital. Patients who had been discharged from the unit to another hospital ward and are re-admitted to the critical care unit are not eligible for re-enrollment into the study.

### Who will take informed consent? {26a}

Under the guidance of the Tri-Council Policy Statement 2, this study has been approved for a waiver of consent (under Article 3.7A) by relevant ethics boards for the following reasons: 4% tetrasodium EDTA is considered at least as safe as the standard of care and has Health Canada approval, there is a strong chance that 4% tetrasodium EDTA will be safer than the standard of care, there have been no adverse outcomes reported in previous clinical use, and the study could not be feasibly completed with a deferred or a full consenting process due to the rapid decision to use a CVAD lock and the need to use it immediately. Due to practical barriers caused by the volume of patients enrolled in the study, it will not be possible for the research staff to inform all patients of their participation in the clinical trial once treatment is finished.

Reminders and signage relevant to the trial will be present in each unit during study procedures. Should patients or caregivers have questions about the trial, a patient/caretaker information letter will be provided along with contact information for the research coordinator. Patients/caretakers will be informed that they can remove themselves or their family members from the trial at any time, with no consequence to their care.

### Additional consent provisions for collection and use of participant data and biological specimens {26b}

This trial does not involve collecting biological specimens for storage (see item {33}).

### Interventions

#### Explanation for the choice of comparators {6b}

The comparator to the trial intervention will be the standard of care CVAD lock. This includes saline for central venous catheters and peripherally inserted central catheters, and citrate for hemodialysis catheters. As the aim of the study is to determine whether 4% EDTA is more efficacious in preventing CVAD complications than the current standard of care, we must compare this alternative to current practices.

#### Intervention description {11a}

Upon admission to the ICU/HAU, any patient with a CVAD requiring locking who meets the criteria will be enrolled in the study. The patient will receive either a standard of care lock or a 4% tetrasodium EDTA locking solution depending on the randomization status of their treating hospital. Syringes containing either 4% tetrasodium EDTA solution or standard of care locking fluid to be used during locking procedures (called “locking kits”) will be prepared ahead of time by the research pharmacy according to the randomization status of the unit. They will be clearly labeled for use as a locking solution only and placed at the patient’s bedside. Syringes will be prepared with an additional label to remind nurses not to use them in pregnant patients or in those who decline any blood products. In addition to the bedside assessment, research coordinators will review each patient that has received a locking solution every day to gather data and to ensure compliance with the study protocols. Nurses will use the prepared syringes to lock and maintain each CVAD according to the standard of care. Syringe supply at each patient’s bedside will be replenished as needed by the research pharmacy and research coordinators. Enrollment can occur 24 h per day, 7 days per week as nurses can obtain locking syringes specific to the randomization status of the unit at all times.

Medication, maintenance fluids, pressors, or anesthesia will be administered through the CVAD according to the standard of care. Once a lumen of the catheter is no longer being utilized for continuous infusions, leftover medication will be aspirated and flushed with 20 mL of 0.9% sodium chloride using a turbulent flush method as per the standard of care. For patients randomized into the experimental group, the catheter will then be locked with the appropriate volume of 4% tetrasodium EDTA, as specified by catheter manufacturer instructions. These procedures will remain exactly the same for patients randomized into the control group with the exception of using saline or citrate as a locking fluid. Experimental and control solutions are indistinguishable by visual inspection.

All further catheter procedures and maintenance will proceed according to the standard of care. For all locked catheters, the frequency of flushing will occur every 12 h (or 72 h for dialysis catheters). During this procedure, catheters locked with the standard of care lock will be aspirated, flushed with 10 mL of 0.9% sodium chloride using turbulent flow, and re-filled with the appropriate volume of the standard of care lock. Procedures will stay exactly the same for patients in hospitals randomized to administer the 4% tetrasodium EDTA treatment, except for continued locking with 4% tetrasodium EDTA. Neutral displacement cap change procedures will remain unchanged in both groups. This includes a change every 96 h, after a blood draw through a cap, if removed, contaminated, damaged, and as needed.

Regular assessments of the site of insertion, CVAD system, and patency will continue according to the standard of care, regardless of the type of locking solution used. According to the adult CVAD maintenance record used at each hospital, nurses will assess that dressing is secure, dry, and intact and will palpate the site and check the system at the beginning of each shift. The site of insertion and connections will be reassessed every 4 h as is standard protocol. Dressing and tubing changes will also proceed according to the standard of care in both groups.

Should a locked line (locked with either standard of care or 4% tetrasodium EDTA solution) needs to be re-opened for additional medication administration, the line will be assessed for patency, aspirated of locking solution, and flushed with 20 mL of 0.9% sodium chloride before administering further medication. Patency will be assessed by the ability to aspirate for blood return and the ability to flush a CVAD without resistance prior to the administration of medications and solutions. If the line is not patent, the nurse will assess for occlusions and proceed with the standard of care occlusion diagnosis and treatment. As per the standard of care, CVADs will continue to be removed at the earliest opportunity for each patient. Four percent of EDTA has been shown to be safe if it is inadvertently flushed into the patient, thereby ensuring an adequate safety profile for use with these vulnerable patients.

#### Criteria for discontinuing or modifying allocated interventions {11b}

The patient may withdraw from the trial at any time. Patients for whom an exclusion criterion is discovered after enrollment will be withdrawn from the study and excluded from the analysis.

#### Strategies to improve adherence to interventions {11c}

The patients themselves do not have to do anything to adhere to the intervention or provide measurements of primary and secondary outcomes. The intervention itself will be administered by the ICU/HAU staff at each center. Strategies to ensure the staff adhere to the experimental procedures include education sessions on the purposes and procedures of the trial before the start of the experiment, and during the trial. The ICU/HAU staff have also been a part of the protocol development to ensure a seamless incorporation of experimental procedures into the standard workflow. The primary and secondary outcomes are already captured as part of the standard of care and will be recorded in patient charts. These charts will be reviewed by the research staff to fill out the study case report forms (CRFs).

#### Relevant concomitant care permitted or prohibited during the trial {11d}

Clinical care will continue as per the discretion of the patient’s healthcare team. The intervention is a CVAD locking fluid that remains only in the lumen of the CVAD; it will never actually enter the patient’s circulation. The locking fluid will be aspirated out of the catheter following the end of locking. As such, there is no potential for drug interactions, and we do not foresee any restrictions in enrolling patients based on other types of concomitant care.

#### Provisions for post-trial care {30}

There is no anticipated harm and compensation for trial participation.

### Outcomes {12}

The following is the primary outcome:

1. Incidence rate of the following: confirmed CLABSI as diagnosed in accordance with the Fraser Health CLABSI diagnosis algorithm (see Additional file 1) based on the NHSN guidelines, CVAD replacement due to occlusion, and/or catheter obstruction requiring alteplase use. The composite incidence rate will be calculated as the number of events divided by the number of catheter days. The patient is considered at risk during the time the patient is in the ICU/HAU and has a catheter placed. Multiple outcomes occurring within a 24-h time frame will be counted as a single event, except for CLABSI; any occurrence less than 2 calendar days following a previous CLABSI and subsequent catheter removal will be considered attributed to the previous catheter, and treated as a single event. The occurrence outcomes will be monitored twice daily via reviewing patient charts and participating with discussions with the patients’ treating nursing staff. Outcomes will be tested for and diagnosed according to clinicians in charge of patient care.

#### Note about the primary outcome

In the case of suspected CLABSI, diagnostic procedures will proceed according to the Fraser Health CLABSI Case Identification Algorithm, in all participating ICU/HAUs. Following CLABSI diagnosis, patients will receive antibiotic treatment as necessary. Should the care team decide that replacement of the CVAD be necessary, treatment will proceed according to the standard of care. Upon subsequent locking procedures, patients will receive the same locking solution as the rest of the hospital unit at that particular point of the study procedure.

The decision to measure CLABSI as an outcome instead of CRBSI was made to keep with the real-world, pragmatic design of the trial. Diagnosis of CRBSI would require specific research-related procurement of samples and microbiological testing that due to the sheer size of the trial, would be too expensive and difficult to implement logistically. Additionally, the use of the CLABSI outcome enables a comparison of rates of catheter-related infections of ICUs participating in this study to those participating in the Canadian Nosocomial Infection Surveillance Program [30]. This surveillance program collects hospital-acquired infection data from 40 participating hospitals across Canada and serves to inform governments and policymakers of the burden of hospital-acquired infections. We chose to also report the same outcome so that the results of our study could be more widely compared to the levels of CLABSI across other Canadian ICUs.

To keep with the current standard of care, CVADs will only be tested for infection at the discretion of the treating physician and will not be tested systematically. This is another factor that we feel justifies the use of CLABSI over CRBSI, although we recognize the outcome is not as rigorous as the CRBSI diagnosis and may be considered a limitation of our work.

#### Secondary outcomes

All outcomes will be assessed twice daily via reviewing the patient charts and participating in discussions with the patients’ treating nursing staff. The outcomes will be tested for and diagnosed according to clinicians in charge of patient care.

1. Incidence rate of confirmed CLABSI (as adjudicated according to the NHSN criteria described previously).
2. Incidence rate of suspected CLABSI. A case of suspected CLABSI will be defined as a case in which no microbiological testing was performed on the suspected catheter or through-catheter blood sample, with the patient having a positive blood culture result of a common bacteria associated with a CVAD infection with no other identifiable source of infection.
3. Incidence rate of catheters requiring removal due to occlusion, as determined by inspecting relevant notes on the patient chart and discussion with the nursing staff.
4. Incidence rate of catheters requiring alteplase use as determined by inspecting relevant notes on the patient chart and discussion with the nursing staff.
5. Incidence rate of catheter-associated venous thrombosis, as determined by inspecting relevant medical imaging and notes on patient charts.
6. Incidence rate of catheter colonization, as diagnosed according to the Clinical Practice guidelines of Infectious Diseases Society of America [31].
7. Classification of types of microorganisms isolated from a convenience sample of removed CVAD’s through culture-dependent and independent techniques.
8. Health economic evaluation of the cost of intervention versus health care utilization and associated costs of diagnosis and treatment of catheter complications

#### Participant timeline {13}

Participant timeline is described in Table 1.

**Table 1:** Participant timeline

Procedure	Pre-lock	First lock	During locking period	During ICU/HAU stay
Informed consent	x^a^			
Inclusion/exclusion criteria	x^b^			
Demographics	x			
Medical history	x			
Consent to receive blood products	x^c^			
Pregnancy test	x^d^			
Prior/concomitant medication	x^e^			
Central line placement (as per SOC)	x			
Central line flushing (q12 as per SOC)^f^		x	x	
Central line aspiration^g^		x	x	
Study medication (in the experimental group)		x	x	
Standard of care (in the control group)		x	x	
Adverse events		x	x	
Data collection	x^h^	x^h^	x^h^	x^i^

^a^Informed consent will be collected in a waived fashion

^b^Confirmation of the eligibility will occur at the baseline visit to make sure that the person still remains eligible

^c^The patient must provide consent to receive blood products before moving forward

^d^Pregnancy test, if completed, would need to be negative before going forward

^e^As other medications may have an unforeseen impact on 4% tetrasodium EDTA, these medications are being tracked to determine if this is the case

^f^Standard of care—the central line is periodically flushed with normal saline or citrate (for hemodialysis catheters) to ensure patency

^g^Standard of care—the central line lock is aspirated of any solution that may remain in the lock between locking or medication administration

^h^Data collection, if applicable, will occur during the defined periods when patients are hospitalized in the ICU/HAU. This data will be collected in order to determine primary and secondary outcomes

^i^Data collection in the form of checking of medical imaging reports will continue for the duration of the patients’ ICU/HAU stay, even if the patient no longer has a central line

Participants will be enrolled during the first three-and-a-half months of each four-and-a-half--month period. Each participant will be followed until they leave the ICU/HAU, they reach the end of the 28-day follow-up period, or they die. Data collection for incidence rates of confirmed/suspected CLABSI, catheter occlusion requiring removal, catheter obstruction requiring alteplase use, direct cost related to alteplase use for catheter occlusion, and catheter colonization will continue via the combination of in-person discussion and chart review for the duration of time that the patient has their catheter in place, until they are discharged from the ICU/HAU, or they die, whichever comes first. In the event that a blood culture is taken on either the last day of catheter placement or the last day that the patient is in the unit, the research coordinator will continue to monitor the patient charts for the result of that blood culture, as a bloodstream infection may still be attributed to the catheter.

Data collection in the form of monitoring medical imaging results for the incidence rate of catheter-associated venous thrombosis will continue for the entirety of the patients’ ICU/HAU stay, even if all CVADs have been removed. The senior investigators’ clinical experience indicates that thrombosis from a CVAD in the upper thoracic region is sometimes not discovered until weeks after the CVAD has been removed, as it may take this long for the patient to develop symptoms of thrombosis. As such, the research coordinators will continue to follow the medical imaging results of the patients who are still in the ICU/HAU but have had all central lines removed and are no longer receiving the study intervention.

### Sample size {14}

The results from a 1-month observational study conducted at Royal Columbian Hospital indicate a 20% baseline risk of the composite outcome [32]. The analysis included central venous catheters, PICC lines, and hemodialysis catheters. In previous clinical studies, 4% EDTA solution has shown a 40–70% reduction in occlusion (measured by total alteplase use before vs. after intervention). Assuming a conservative 30% relative risk reduction in the risk of the composite outcome, our sample size calculation is based on an absolute risk reduction from 20 to 14%. A total of 1690 patients need to be recruited to achieve adequate power.

The simulated sample size calculations were performed using R with 10,000 simulation runs, which yields a standard error of 0.4% in the estimated power. The calculation assumed a standard deviation of baseline risk across different ICUs to be 0.02, which corresponded to a within-cluster within-period correlation of 0.0029. The standard deviation between cluster time periods was assumed to be 0.001. A generalized linear mixed effect model was used to generate and analyze the data. The statistical significance was determined using a *t*-test with degrees of freedom equal to the number of cluster periods minus the number of periods minus one [33].

### Recruitment {15}

The results of the observational study conducted at Royal Columbian Hospital’s 30-bed ICU and step-down unit in December 2020 reveal that 75 patients out of a total of 90 met the inclusion criteria to be enrolled in the study. Conservatively, we aim to recruit at least 50 patients per unit per month at each participating unit. We do not perceive recruitment challenges as we have obtained waived consent from relevant ethics boards.

With a three-and-a-half-month enrollment window during each period, each site is expected to recruit at least 175 patients per period. As five ICUs will be participating in this study and each site enrolls for two periods, the enrollment will be 1750 patients in total. Protocol adherence will be deemed a success if there is greater than 85% of patients who are adherent to the entire study protocol.

### Assignment of interventions: allocation

#### Sequence generation {16a}

Randomization involves only the allocation of the five ICUs to the locking solution that will be used during the first period. The randomization will be performed by a statistician at the Centre for Health Evaluation and Outcome Services (CHEOS).

#### Concealment mechanism {16b}

The statistician performing the randomization will disclose the allocations to only the study project manager (PM). The PM will then liaise with each site pharmacy to inform them of their experimental condition so that they can prepare the appropriate locking kits during each period. Blinding of clinicians and research staff will remain throughout the entirety of the trial.

#### Implementation {16c}

Allocation sequence will be generated by the external statistician. Study participants will be enrolled by designated research coordinators at each site. All patients at each participating site will receive the treatment which is being administered at the site during that period.

### Assignment of interventions: blinding

#### Who will be blinded {17a}

Trial participants, care providers, outcome assessors, and data analysts will be blinded to the assignment of intervention. The only research personnel who will be unblinded are the statistician performing the randomization, the PM, and the research pharmacists in charge of supplying locking kits used during the study. As each of the 4% tetrasodium EDTA, saline, and citrate will be placed in 10mL syringes, they will not be distinguishable to the trial participants, care providers, or outcome assessors to maintain their blinding. Data analysis will occur outside of the unit with the type of intervention received by each patient concealed until the completion of analysis.

#### Procedure for unblinding if needed {17b}

If unblinding is necessary for patient management (e.g., in the case of an adverse event (AE) for which patient management requires knowledge of treatment assignment), the investigator will be able to obtain this information by getting approval from the study principal investigator (PI) to contact a designated individual with access to the allocation list. The study PI and the designated individual will be available 24 h per day and 7 days per week. Treatment codes will not be broken except in emergency situations that affect clinical care decisions. The investigator requesting unblinding will document and provide an explanation for the request.

### Data collection and management

#### Plans for assessment and collection of outcomes {18a}

All information collected for each patient will be obtained either by reviewing patient charts or engaging in conversations with the treating nurses daily. Members of the research team at each site will extract each participant’s study data from the patient charts and/or resulting nursing conversations and enter the data directly into a secure, electronic database. The lead investigator will instruct each of the study sites regarding data capture procedures on electronic and/or paper CRFs.

#### Plans to promote participant retention and complete follow-up {18b}

All experimental procedures will be administered by ICU/HAU and the research staff during the patient stay. As such, there will be no efforts to promote participant retention.

Patients will be followed by the members of the research team until they leave the ICU/HAU, they reach the end of the follow-up period, or they die. After leaving the ICU/HAU, the patients will no longer be part of the study and will not be retained. Patients who are transferred out of the ICU/HAU with a central line in situ will no longer receive the study locking solution and revert to the standard of care for the unit to which they have been transferred to. To avoid logistical complications due to the sheer number of participants, patients who leave the ICU/HAU for another hospital unit with their original CVAD still inserted will not be followed. Patients will not be followed past the end of the experimental period.

#### Data management {19}

Data will be managed by an external data monitoring center. The secure database will be managed according to the standard operating procedures and will remain in secure locations on site throughout the entirety of the study.

#### Confidentiality {27}

In order to maintain patient privacy, data capture tools, study drug accountability records, study reports, and communications will identify the assigned patient number. Confidentiality standards are maintained by coding each patient enrolled in the study through the assignment of a unique patient identification number. Patient names or any identifying information will not be included in the aggregate data that are transmitted for analysis. Only site enrollment logs maintained by research coordinators in charge of data acquisition will contain any personal identifiers. These logs will remain in a secure location on site for the duration of the study. Only research coordinators will access this information. Additionally, the site investigators will grant monitor(s) and or its designee access to the patient’s original medical records, including medical history, laboratory studies, and medication administrations, for verification of data gathered and auditing the data collection process. This information will be accessed for the duration of the study for the purpose of data verification and reconciliation.

#### Plans for collection, laboratory evaluation, and storage of biological specimens for genetic or molecular analysis in this trial/future use {33}

A sample of convenience (*n* = 15) of all catheters removed for suspected or confirmed CLABSI or colonization during work hours at the lead investigators site will be collected and used for the analysis of biofilm formation and classification of microorganisms. The same number of CVADs removed from patients who no longer require a CVAD with the absence of colonization will also be collected to act as a control comparator in this analysis. Upon removal, these CVCs will be placed in specimen bags stored at 4 °C. These catheters will then be processed by study personnel before shipment to Dr. Aaron White’s lab at the Vaccine and Infectious Disease Organization-International Vaccine Centre (VIDO-InterVac) for further analysis. These will include culture-dependent and culture-independent classification of microorganism colonies and analysis of biofilm, according to internal standard operating procedures.

## Statistical methods

### Statistical methods for primary and secondary outcomes {20a}

Data will be analyzed following the intent-to-treat principle. The primary outcome will be analyzed using a zero-inflated negative binomial (ZINB) model that accounts for clustering within ICU [33]. The ZINB model takes into account: (1) the large percentage (~ 80% expected) who will not have any event and (2) the large variability in the number of events among participants who experience at least one event (individuals who experience one event are more likely to experience additional events). The (log-)time-at-risk will be entered into the model as an offset term. The average treatment effect will be obtained by regression standardization of the composite incidence rates predicted by the ZINB model [34]. Standard errors will be obtained using bootstrapping.

The model will adjust for the study period (first or second) and may adjust for the following individual-level baseline characteristics: age; sex at birth; BMI; APACHE IV score; length of pre-ICU hospital stay (days); presence of multiple CVADs per patient; any suppression of the immune system, defined as post-organ transplantation, acquired immunodeficiency syndrome [AIDS], neutropenia [< 1000 absolute neutrophils], corticosteroids [> 20 mg/day of prednisone or equivalent for more than 6 months], or currently taking immunosuppressive agents (i.e., Imuran, etc.) at time of enrollment into study; site of CVAD insertion (femoral, jugular, subclavian, right or left side); type of catheter used; any catheter additives, as they may confound the result (such as antibiotic impregnation); having a pre-existing central line upon admission to the ICU; and this line had already received a lock, concurrent systemic antibiotic use unrelated to central line infection, concurrent systemic anti-fungal agent use unrelated to central line infection, concurrent systemic administration of anti-coagulants unrelated to catheter occlusion, type of hemodialysis (if applicable), and previously experienced CLABSI or CVAD occlusion.

The secondary outcomes will be analyzed similarly to the primary analysis using generalized mixed effects models (to account for clustering within the unit) appropriate to the type of outcome. Further analysis of the results will include the calculation of costs attributed to healthcare utilization costs between the groups. A complete statistical analysis plan will be developed prior to unblinding and analysis of the data.

### Interim analyses {21b}

Due to the cluster-crossover design, there will be no sufficient data from both treatment conditions to enable the within-center comparisons until near the end of the study. As such, interim analysis for early efficacy or futility would not be informative. Preliminary data will be analyzed only for safety (by tabulating the total percentage of AEs observed) and recruitment rates (by reviewing the total number of patients enrolled and comparing this to predicted rates).

### Methods for additional analyses (e.g., subgroup analyses) {20b}

Subgroup analysis will be conducted for each participating ICU to assess the potential heterogeneity of treatment effects across sites using a generalized linear model stratified by ICU. Additionally, due to standard of care locking differing between CVCs, IVADs, and PICCs (locked with saline) as compared to hemodialysis catheters (locked with citrate), the authors will perform an analysis of the hemodialysis population both together and separately from the rest of the patient population.

During the COVID-19 pandemic, the expansion and shuffling of ICU and HAU patients to different units of the hospital pose a logistical problem in running experimental protocol (as this would involve having to provide education and oversight of nursing staff beyond the ICU/HAUs). As such, patients who are still designated as ICU/HAU care level, but physically located in another unit, will not receive the locking intervention and will be followed by Research Coordinators for their location in the study. Should they be repatriated back to the physical spaces of the ICU/HAU, they will resume receiving the study locking protocol. Because these patients would have experienced an interruption to the experimental protocol, they will also be analyzed in a separate subgroup.

Finally, recent studies have also provided evidence of a higher risk of catheter occlusion and catheter-associated venous thrombosis among COVID-19 patients, due to the hyper-inflammation and hyper-coagulation associated with the virus [35–37]. As such, we will be collecting data about the COVID-19 status of each enrolled patient. This subgroup will also be analyzed separately, to determine if there are any differences among the COVID-19 population.

### Methods in analysis to handle protocol non-adherence and any statistical methods to handle missing data {20c}

We do not expect any issues related to protocol non-adherence as detailed education about trial procedures will be given to all the clinical staff involved prior to the study start. No cross-over is expected; only one of the two locking solutions will be available at each site during each period.

We do not expect to have a significant number of missing data points as we are not including any measures that need to be collected outside of the standard of care. Multiple imputation will be used to fill in missing data on covariates, if needed. Should any outliers arise, we will conduct a sensitivity analysis to assess their impact on the conclusions.

### Plans to give access to the full protocol, participant-level data, and statistical code {31c}

Participants may request access to de-identified data. Patient-specific data will be available at the completion of data analysis.

### Oversight and monitoring

#### Composition of the coordinating center and trial steering committee {5d}

The daily operations of this project will be managed by site research coordinators. Each center will appoint a research coordinator responsible for enrollment and daily data collection, as well as recording of AEs. Any AEs and enrollment numbers will be communicated to a separate trial steering committee on a bi-weekly basis by each site research coordinator. The trial steering committee will consist of co-investigators and will meet via teleconference on a bi-weekly basis, starting at 4 weeks prior to the study start to discuss trial progress and any necessary protocol amendments. The last meeting will be held 4 weeks after the study end. The trial steering committee will also be responsible for producing the final paper for publication. The data collection database will be according to the internal standard operating procedures.

#### Composition of the data monitoring committee, its role, and reporting structure {21a}

Four percent of EDTA solution has a very low possibility of harm as it is already Health Canada approved, is marketed, and is sold, and patients have minimal systemic exposure as it will be aspirated prior to the lumen being used. As such, investigators feel a standing DMC is not necessary. If at any time, more than 3 serious adverse events are recorded, a DMC will be assembled to review the trial safety.

#### Adverse event reporting and harms {22}

Information about any AEs will be collected with routine data collection. AEs will include (1) local site irritation or redness following accidental administration of the catheter lock into the patient circulation, (2) dysgeusia following accidental administration of the catheter lock into the patient circulation, (3) paresthesia following accidental administration of the catheter lock into the patient circulation, (4) pain upon accidental administration of the catheter lock into the patient circulation, (5) “head rush” following administration, (6) blood rush to face and lips following administration, (7) hypocalcemia due accidental administration of the catheter lock into the patient circulation, and (8) any other adverse reaction observed during accidental administration of the catheter lock into the patient circulation.

#### Frequency and plans for auditing trial conduct {23}

Monitoring and auditing procedures developed by the lead site investigator, or its designee, will be followed, in order to comply with the Good Clinical Practice and International Council for Harmonization of Technical Requirements for Pharmaceuticals for Human Use (ICH) guidelines. Inspection of the database for consistency completeness and clarity, in comparison with source documents, will be performed. Any clarification of administrative matters will also be completed in this process. The study will be monitored by the lead site or its designee. Monitoring will be done by personal or virtual visits from a representative of the lead site.

All AEs will be reviewed by the principal investigator. All AEs will be reported to the relevant ethics boards within 72 h of their occurrence. The relevant ethics boards will perform audits as per standard operating procedures. The independent physician and members of the relevant ethics boards are both independent from the investigators and the sponsor.

#### Plans for communicating important protocol amendments to relevant parties (e.g., trial participants, ethical committees) {25}

Research coordinators and investigators at each site will meet on a bi-weekly basis to discuss ongoing trial progress. Any important protocol modifications will be discussed and communicated during these meetings. Study materials will also be updated with any new information and given to patients and caretakers as necessary. Any changes to the protocol must be approved by the relevant ethics boards prior to implementation.

To keep the public informed on clinical trials in a timely manner, and to comply with applicable laws, regulations, and guidance, this study is registered with ClinicalTrials.gov. This entry will be maintained by the members of the research team to always display up-to-date information about the trial.

#### Dissemination plans {31a}

Completion and results from this study will be used to fulfill thesis requirements for one graduate-level student. Plans for publication include submission of the thesis, publication in an academic journal of the student/supervisor choosing, and oral dissemination of results in the form of a formal thesis defense as well as submission to conference proceedings. Conference presentations will serve to disseminate results to academic and clinical professionals.

In addition, patient partners will be engaged to identify target audiences outside of the medical research community and direct the creation of meaningful knowledge dissemination materials for these audiences, such as infographics, brochures, or digital media.

If successful, the results of this trial can be used to inform key decision-makers of available improvements to CVAD practices and ICU budgets through the use of 4% tetrasodium EDTA. These improvements may lead to changes to the standard of care to include novel CVAD lock solution technology.

## Discussion

With regard to the decision to exclude readmissions, results from the 1-month pilot study at the lead site indicate re-admission is rare (< 2% of admissions), so their exclusion has a negligible impact on the study results.

Anecdotal evidence gathered from 4% tetrasodium EDTA manufacturers (SterileCare Inc.) acknowledges that aspiration of the standard of care locks prior to flushing procedures or medication administration is not usually done in everyday practice; the locking fluid from the CVAD is flushed into the patient circulation. This poses no harm to the patient. As 4% tetrasodium EDTA solution is only approved by Health Canada as a locking solution, and not as a flush, these procedures will have to change during study operation and nurses will have to aspirate all locked CVADs every 12 h (or 72 h for hemodialysis catheters). This will be included in the education of ICU staff prior to the procedure start.

Finally, multiple crossover events were considered while designing the trial protocol [36]. The benefit of multiple crossovers includes the potential of higher statistical power. This was cautiously weighed against an increase in the experimental timeline due to the increased number of follow-up periods with multiple crossover events. Due to considerations of time and funding, the authors have opted to perform a study with only one crossover event.

## Trial status

The finalized protocol version is 2.5, May 2022. Recruitment at the lead site began in March 2022. The approximate date when recruitment will be completed at all sites is August 2023.

## Supplementary Information

**Additional file 1.** Fraser Health CLABSI Case Identification Algorithm.

## Data Availability

The investigators will retain control over the data. De-identified data may be available from the investigators at the conclusion of the trial.

## Abbreviations

CVAD: Central venous access device
CLABSI: Central line-associated bloodstream infection
ICU: Intensive care unit
EDTA: Ethylenediaminetetraacetic acid
PICC: Peripherally inserted central catheter
HAU: High acuity unit
TSDM: Temporary substitute decision-maker
CRFs: Case report forms
ICC: Intraclass correlation coefficient
CHEOS: Centre for Health Evaluation and Outcome Services
PM: Project manager
PI: Principal investigator
AE: Adverse event
VIDO: Vaccine and Infectious Disease Organization
APACHE: Acute Physiology And Chronic Health Evaluation
DSMB: Data Safety Monitoring Board
ICH: International Council for Harmonization of Technical Requirements for Pharmaceuticals for Human Use
