# Supplementary material for: Control of Line Complications with KiteLock (CLiCK) in the critical care unit: study protocol for a multi-center, cluster-randomized, double-blinded, crossover trial investigating the effect of a novel locking fluid on central line complications in the critical care population
Source: Trials. 2022 Aug 30;23:719. doi: 10.1186/s13063-022-06671-5 (PMC9425798; doi:10.1186/s13063-022-06671-5)
Supplement: Supplementary file 1 — Additional file 1. Fraser Health CLABSI Case Identification Algorithm. [file 13063_2022_6671_MOESM1_ESM.pdf]

Fraser Health CLABSI Case Identification Algorithm

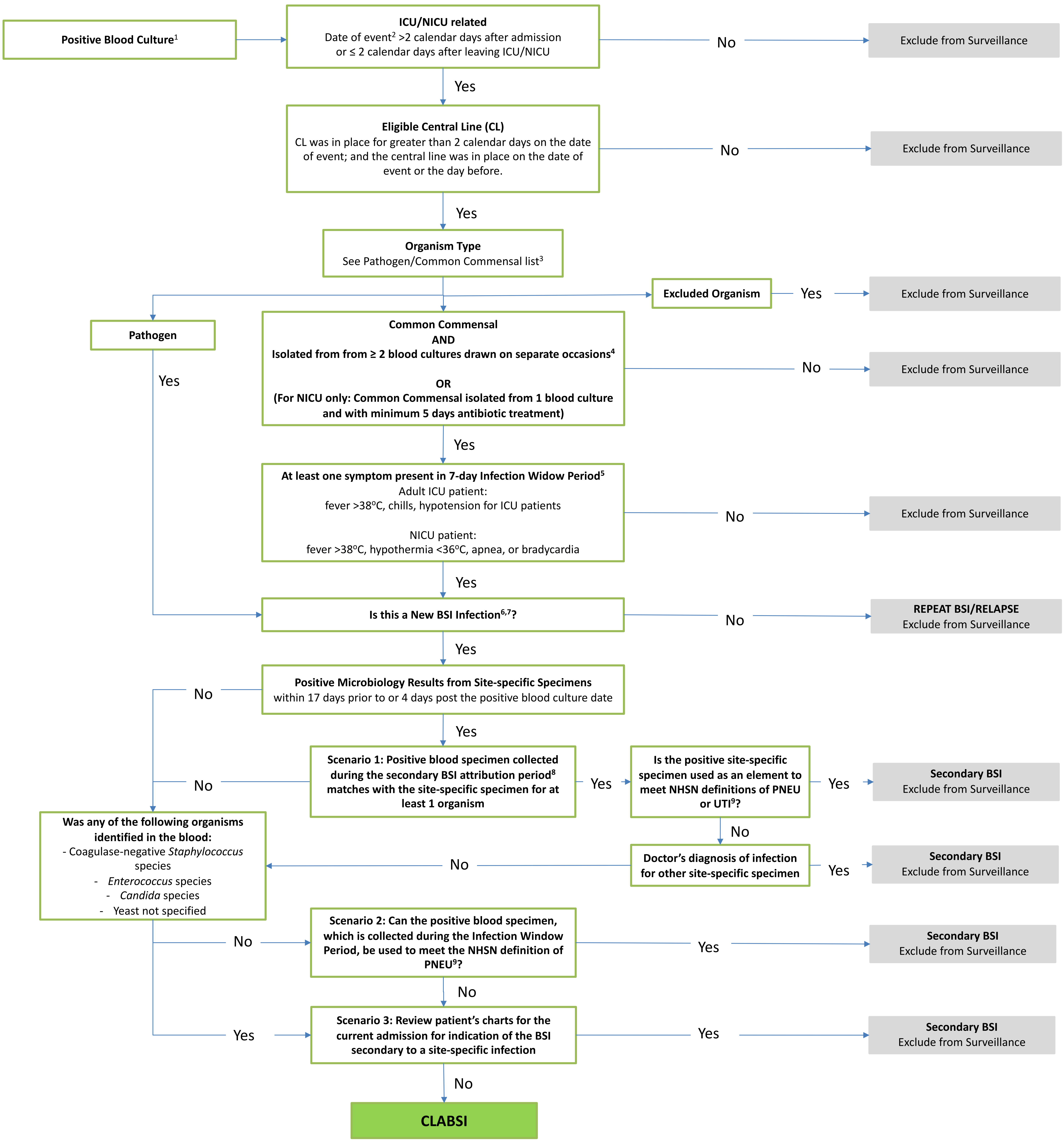

1. **Positive Blood Cultures:** identified by pulling Meditech report "Positive Blood Cultures – ICU".
2. **Date of Event (or onset date):** the date the first element used to meet the BSI criteria occurs for the first time within the seven-day infection window period.
3. **See Pathogen/Common Commensal list** here.
4. **Separate occasions:** Blood from at least two separate blood draws were collected on the same or consecutive calendar days and two separate site preparations were performed during specimen collection.
5. **Infection Window Period:** 7 days during which all site-specific infection criteria must be met. It includes the day of the first positive diagnostic test (for BSI, it is the date of the first positive blood culture), the 3 calendar days before and the 3 calendar days after.
6. **New BSI vs. Relapse:** Same microorganism (as best as can be determined by the data available – e.g., species, antibiotic sensitivity) isolated from a subsequent blood culture
   - If ≤10 days from a negative culture OR ≤10 days from completion of appropriate antibiotic therapy, consider as a relapse and Do Not Report.
   - If >10 days from a negative culture AND >10 days from completion of appropriate antibiotic therapy, REPORT as a new infection.
7. **New BSI vs Repeat BSI:** If BSI case definition is met and the date of event is within the 14-day Repeat Infection Timeframe of a previous primary BSI, consider as repeat and Do Not Report.
8. **Secondary BSI Attribution Period:** The infection window period of the primary infection event combined with the Repeat Infection Timeframe.
9. **NHSN definitions:** refer to the NHSN definitions of PNEU and UTI.
